# Surface electromyography study on asymmetry in paravertebral muscle degeneration in patients with degenerative lumbar scoliosis

**DOI:** 10.1038/s41598-022-23541-x

**Published:** 2022-11-04

**Authors:** Hongru Xie, Jianan Liu, Yinchuan He, Zepei Zhang, Hongtao Dong, Lin Meng, Jun Miao

**Affiliations:** 1 Graduate School, Tianjin Medical University, No. 22 Qixiangtai Road, Heping District, Tianjin, 300210 China; 2 Department of Spine Surgery, Tianjin Hospital of Tianjin University, No. 406 Jiefang South Road, Hexi District, Tianjin, 300210 China; 3 Academy of Medical Engineering and Translational Medicine, Tianjin University, No. 92 Weijin Road, Nankai District, Tianjin, 300210 China; 4 Yuncheng Central Hospital, No. 3690 Hedong East Road, Yuncheng, 044000 Shanxi China

**Keywords:** Computational biology and bioinformatics, Medical research

## Abstract

The asymmetry of paravertebral muscle (PVM) degeneration in degenerative lumbar scoliosis (DLS) patients has been extensively studied by imaging and histological examination and has not yet been verified by surface electromyography (sEMG) techniques. To study the relationship between the surface electromyography (sEMG) and degenerative characteristics of paravertebral muscles (PVMs) in patients with degenerative lumbar scoliosis (DLS). In twenty DLS patients and fifteen healthy subjects, sEMG activity of the PVMs at the level of the upper end vertebra (UEV), apical vertebra (AV) and lower end vertebra (LEV) was measured during static standing and dynamic standing forward flexion and backward extension tasks. Action segmentation was achieved according to inertial measurement unit (IMU) data. The sEMG characteristics of the PVMs on the convex and concave sides were compared, and the relationship of these data with the Cobb angle and lumbar lordotic angle (LL) was analyzed. In the DLS group, there was no difference in sEMG activity between the convex and concave sides at the UEV or AV level, but in the motion and return phases of the standing forward flexion task (P = 0.000, P = 0.015) and the maintenance and return phases of the standing backward extension task (P = 0.001, P = 0.01), there was a significant difference in sEMG activity between the convex and concave sides at the LEV level. Asymmetrical sEMG activity at the LEV level was negatively correlated with the Cobb angle (F = 93.791, P < 0.001) and LL angle (F = 65.564, P < 0.001). In the DLS group, asymmetrical sEMG activity of the PVMs appeared at the LEV level, with the concave side being more active than the convex side. This sEMG characteristics were consistent with their imaging and histological degenerative features and correlated with bone structural parameters.

## Introduction

Degenerative lumbar scoliosis (DLS) is a spinal deformity in which the lumbar spine degenerates after skeletal maturity, resulting in scoliosis with a Cobb angle > 10° on the coronal plane that is often accompanied by a change in sagittal balance. It has a complex pathogenesis, a long course, and a progressive trend.

Previous studies have demonstrated the association between paravertebral muscle (PVM) degeneration and spinal disorders and the prevalence of PVM degeneration in DLS patients^1,2^. Therefore, the study of PVMs in the lumbar spine, especially asymmetry in PVM degeneration, is important to further understand the occurrence and development of lumbar degenerative diseases. In this regard, domestic and foreign scholars have conducted a large number of verification studies through imaging and histological examinations^3–5^.

Electromyography (EMG) is a recording of neuromuscular activity, and its characteristics represent changes in muscle function and can be used to analyze the relationship between PVMs and scoliosis^6^. The surface EMG (sEMG) characteristics of PVMs in the dynamic state are valuable in clinical evaluations, and EMG changes in subjects during dynamic movements can reflect the function of the neuromuscular system^7,8^.

At present, most sEMG studies have focused on AIS patients, and there have been few related studies on degenerative scoliosis. In this study, sEMG was used in combination with inertial measurement units (IMUs) to examine sEMG changes in PVMs in DLS patients during static standing and dynamic standing forward flexion and backward extension movement tasks. By measuring the sEMG data of PVMs at multiple levels (upper end vertebra (UEV), apical vertebra (AV), and lower end vertebra (LEV)), we were able to compare the convex and concave sides and analyze correlations between these data and bone structural parameters. The purpose was to summarize the relationship between the sEMG characteristics of PVMs and the degree of PVM degeneration in DLS patients.

To the best of our knowledge, this is the first study to combine sEMG with the use of IMUs to record the sEMG activity of multiple PVMs simultaneously during motion in DLS patients, verify asymmetry in activity and analyze degenerative characteristics by convex-concave side comparisons.

## Methods

### Subjects

The subjects included twenty DLS patients diagnosed in Tianjin Hospital from March 2021 to September 2021. The inclusion criteria were as follows: (1) no history of lumbar scoliosis, diagnosis of lumbar scoliosis upon examination, and Cobb angle > 10°; (2) age > 50 years; (3) routine imaging examination, including full-length spine view; and (4) no nerve root lesions in untreated patients. The control group consisted of fifteen healthy persons who underwent a comprehensive physical examination at Tianjin Hospital from March 2021 to September 2021. The inclusion criteria were as follows: (1) age > 50 years; (2) no degenerative lumbar spine disease; and (3) no radiculopathy or treatment.

The medical ethics committee of the hospital approved the study, and informed consent was obtained from the patients themselves and their families in all cases. All experiments were performed in accordance with the relevant guidelines and regulations, i.e. the reports in the manuscript follow the recommendations in the Declaration of Helsinki.

There were no significant differences in sex, age, height, weight, or body mass index (BMI) between the two groups (Table 1).

**Table 1:** Demographic characteristics of the control group and DLS group (mean ± SD).

Variable	Control (N = 15)	DLS (N = 20)	Statistics	P value
Gender (F/M)	9/6	14/6	χ^2^ = 3.55	0.169
Age (years)	63.67 ± 7.24	63.2 ± 6.67	t = − 0.647	0.528
Height (m)	1.66 ± 0.06	1.63 ± 0.04	t = − 1.744	0.103
Weight (kg)	73.10 ± 9.17	67.53.0 ± 8.25	t = − 1.364	0.194
BMI	26.81 (17.63–29.39)^a^	25.49 ± 3.22	Z = − 1.079	0.281

^a^The BMI of the control group showed non-normal distribution, and the median was used to describe the data.

The exclusion criteria were as follows: (1) significant limitation of standing and lumbar extension and flexion activities; (2) manifestation of lower limb neuralgia; (3) abnormal vertebral development, spinal fracture and spinal instability seen in imaging data; and (4) spine, pelvis, and lower limb surgery history.

### Imaging measurements

**Figure 1:**
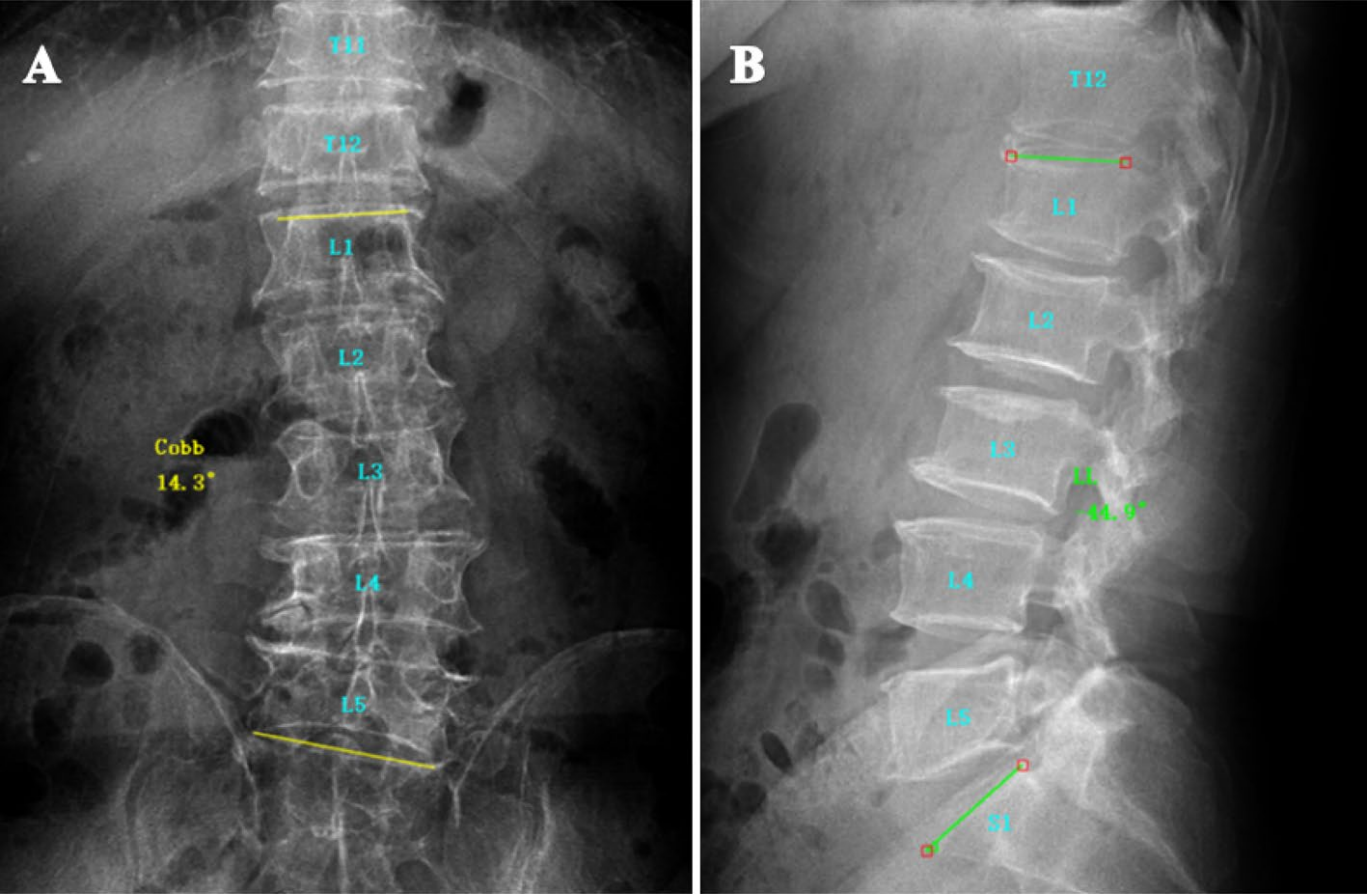
Male, 75 years, DLS with left-sided convexity. (**A**) The UEV was the L1 vertebra, the AV was the L3 vertebra, the LEV was the L5 vertebra, and the Cobb angle was 14.3°. (**B**) The LL angle was 44.9°.

In the DLS group, the Cobb angle between the upper endplate of the UEV and the lower endplate of the LEV on the coronal plane view of the lumbar spine was measured with standard full-length anteroposterior radiography, and the lumbar lordotic (LL) angle between the L1 upper endplate and the S1 upper endplate was measured in standard lateral radiography. All data were analyzed using Surgimap software (Version 2.3.2.1; National Institutes of Health; USA). The measurement results were as follows: In the DLS group, fourteen samples had left-sided convexity, six samples had right-sided convexity, the average Cobb angle was 17.6° ± 5.20°, and the average LL angle was 32.84° ± 8.96° (Fig. 1).

### Electromyographic recording

**Figure 2:**
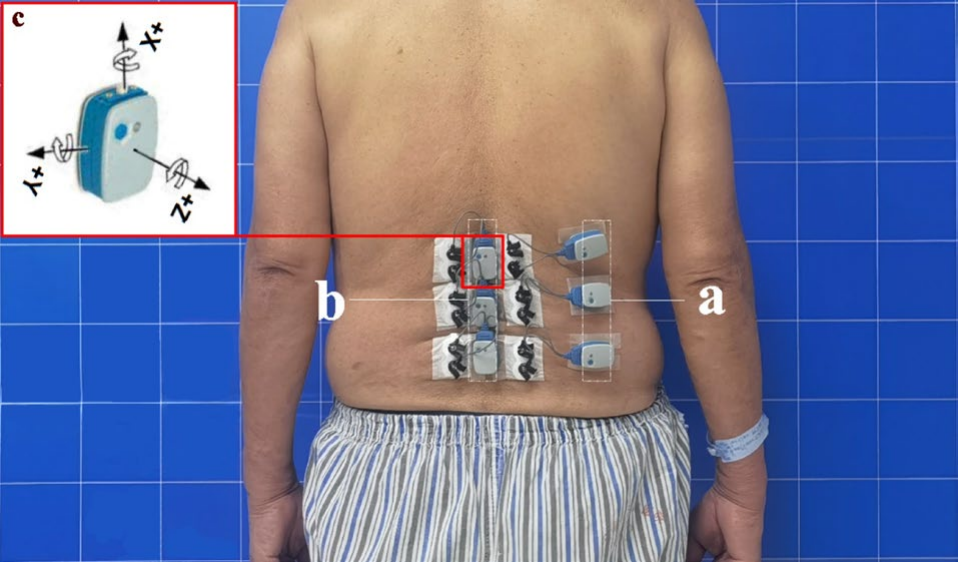
Six bipolar electrodes were placed at three levels of the bilateral PVMs (UEV, AV and LEV). After confirmation by radiographs, the sEMG sensors and IMUs were placed in the appropriate areas. (a) Three sEMG sensors on the right. (b) Three sEMG sensors on the left, each integrated with an IMU, placed on the corresponding position of the spinous process (L1, L3 and L5). (c) Three axes of the IMU.

The NORAXON™ ultium EMG Biomechanics system (Noraxon, USA, Inc.) was used to record sEMG signals at a sampling frequency of 2000 Hz. According to the European sEMG recommendations^9^, after skin preparation, Ag/AgCl (Shanghai, China) bipolar circular surface electrodes (44 × 22 mm; electrode spacing, 18 mm) prepared with conductive gel were placed on the abdomen of bilateral PVMs at the level of the UEV, AV and LEV (Fig. 2), while the control group was placed at the level of L1, L3 and L5, consistent with the direction of muscle fibers^10,11^.

### Tasks and procedures

**Figure 3:**
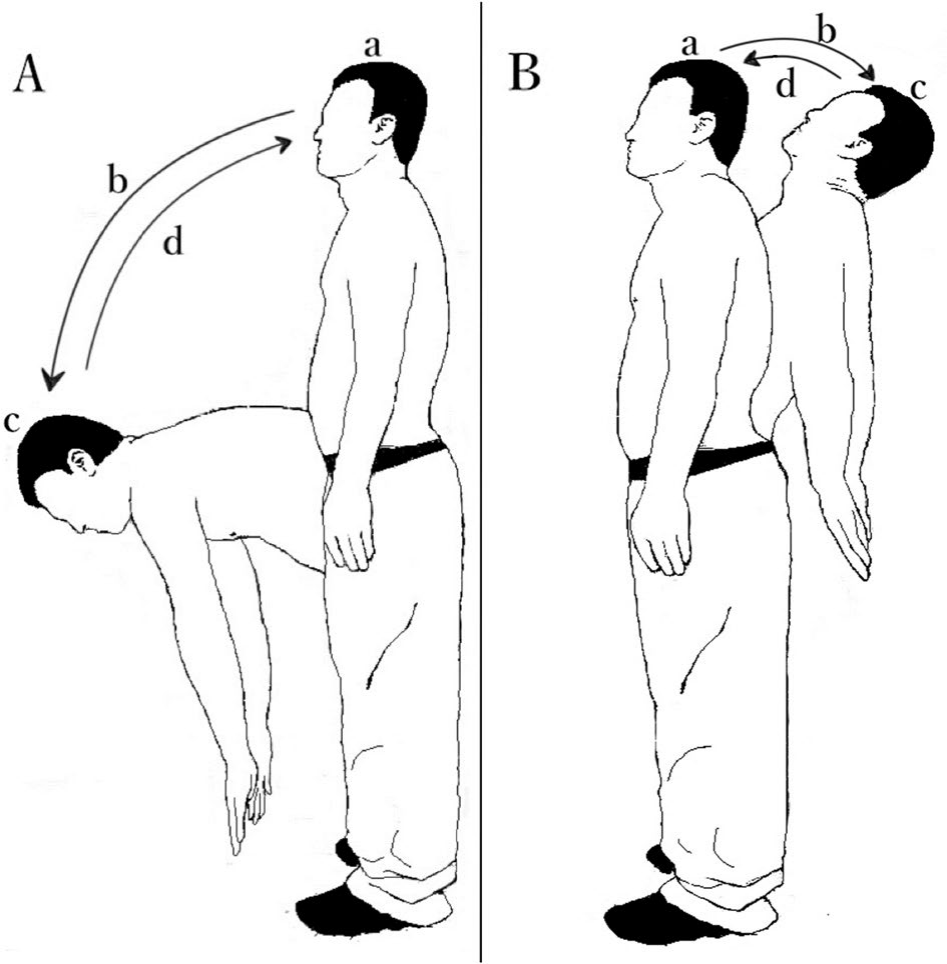
A subject performs movement tasks. (**A**) Standing forward flexion task. (**B**) Standing backward extension task. (a) Static standing phase. (b) Motion phase of the standing flexion/extension tasks. (c) Maintenance phase of the standing flexion/extension tasks. (d) Return phase of the standing flexion/extension tasks.

Movement tasks included maximum standing forward flexion and maximum standing backward extension. Under the guidance of the metronome, subjects were instructed to (a) static standing for 5 s, (b) perform maximum forward flexion/backward extension of the trunk at a self-selected speed for 5 s, (c) remain in forward flexion/backward extension for 5 s, and (d) return to the initial static standing position for another 5 s (Fig. 3). Each movement phase was performed at a uniform speed.

sEMG signals during trunk forward flexion and backward extension movements were analyzed, and the sEMG signals from phase (a) were used as the static standing phase. Each patient was tested three times, and the average of the three tests was used for further analysis.

Before the formal measurements, participants had a practice session to familiarize themselves with the experimental procedure, and patients were instructed to perform lumbar extension and flexion activities, avoiding pelvic and lower limb involvement as much as possible. To prevent fatigue, there was a 1-min break between any two consecutive tests.

### IMU

**Figure 4:**
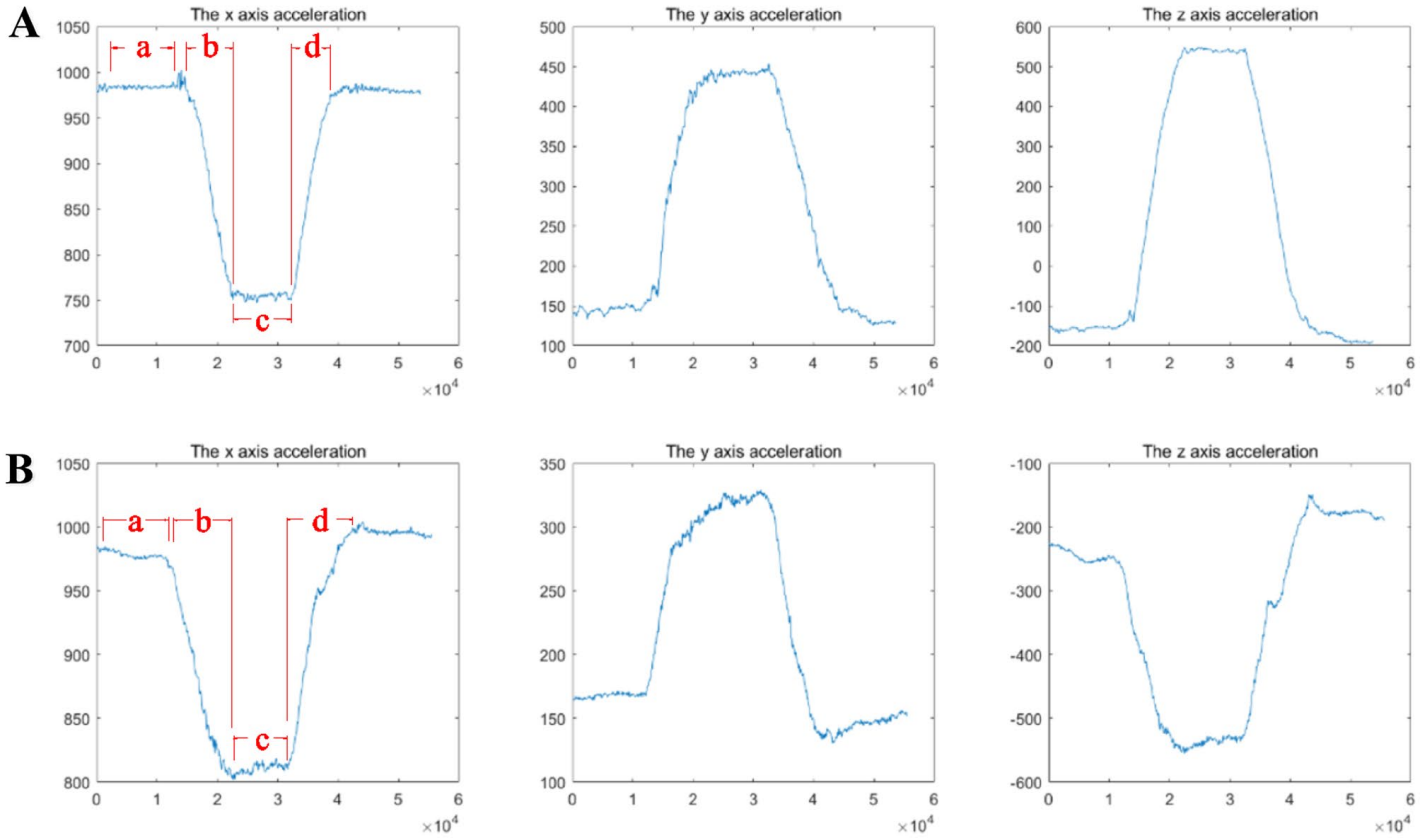
Typical images of the three sets of signals collected with the IMU after processing: acceleration along the vertical axis (x-axis), horizontal axis (y-axis), and anterior–posterior axis (z-axis). (**A**) Standing forward flexion task. (**B**) Standing backward extension task. (a) Static standing phase. (b) Motion phase of the standing flexion/extension tasks. (c) Maintenance phase of the standing flexion/extension tasks. (d) Return phase of the standing flexion/extension tasks. The x-axis is the data collection frequency (Hz); the y-axis is the acceleration of the movement (mg).

The three sEMG sensors on the left were each integrated with an IMU^12–15^ and fixed to the L1, L3, and L5 horizontal spinous processes using double-sided adhesive, allowing measurement of the angular velocity of lumbar spine motion. Motion signals from three IMUs were collected: acceleration along the vertical axis (x-axis), along the horizontal axis (y-axis), and along the anterior–posterior axis (z-axis) (Fig. 4). sEMG and IMU data acquisition was performed simultaneously.

### sEMG signal processing

**Figure 5:**
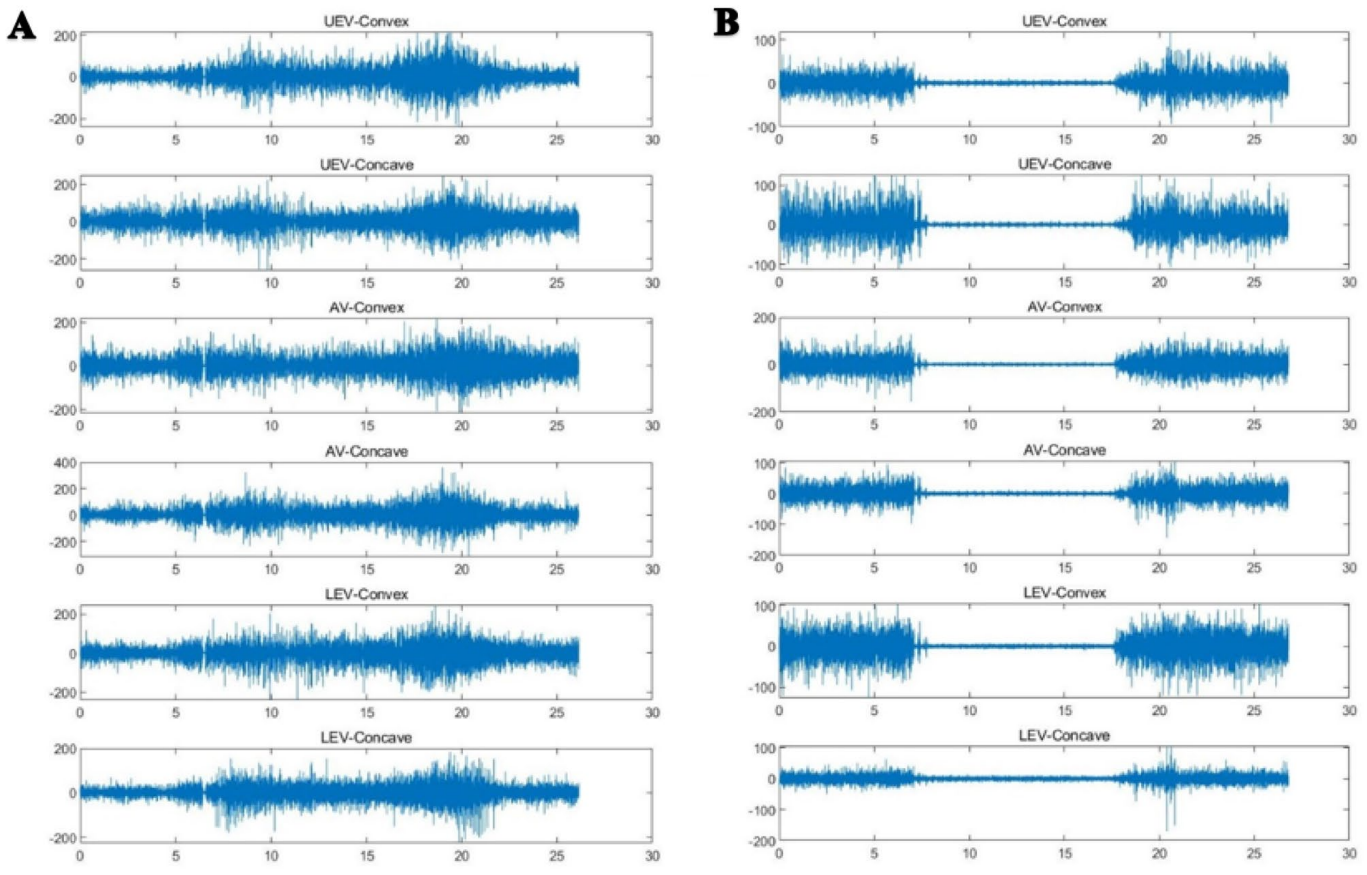
Typical sEMG signal of the subject's PVMs after pretreatment on the convex/concave sides at the three measurement levels. (**A**) Standing forward flexion task in DLS group. (**B**) Standing backward extension task in DLS group. The x axis is the sampling time (seconds), and the y axis is the EMG amplitude (uV).

The original sEMG signals were processed with a Butterworth bandpass filter (30–500 Hz), and the baseline noise was removed (Fig. 5). The starting and ending points of each motion phase in the movement task were segmented and identified with the kinematic data collected synchronously by the IMU; incorporating smoothed three-second data trials for each motion phase (Fig. 4). The root mean square (RMS) was calculated with MATLAB software (MATLABR2020a, MathWorks, Natick, MA, USA) and normalized to the ratio of the maximum voluntary contraction (MVC)^16^.

### Statistical analysis

Statistical analysis was performed using SPSS 25.0 (IBM Corporation, Armonk, NY, USA). The measurement data are described by the mean ± SD. All data were analyzed by descriptive analysis and normality tests. A nonparametric test was used to compare the demographic data of the two groups. Paired *t* tests were used to compare the differences in bilateral PVMs (left/right or convex/concave). A simple linear regression model was used to analyze the influence of Cobb angle and LL angle on the convex/concave sEMG ratio at the LEV level. By drawing the scatter plot, we can judge whether there is a linear relationship between them. P < 0.05 was considered a statistically significant difference.

The study protocol was approved by the ethical review committee of Tianjin Hospital, and all participants and their legal representatives gave informed consent to participate in the study.

### Ethics approval and consent to participate

The study was approved by the Ethical Committee of Tianjin Hospital (2020088). The participants provided written informed consent.

## Results

The sEMG values (mean ± SD) and statistical analysis results for the three levels of PVMs (L1/L3/L5 or UEV/AV/LEV) during the three movement tasks (static standing, standing forward flexion, and standing backward extension) for the DLS and control groups are presented in Table 2.

**Figure 6:**
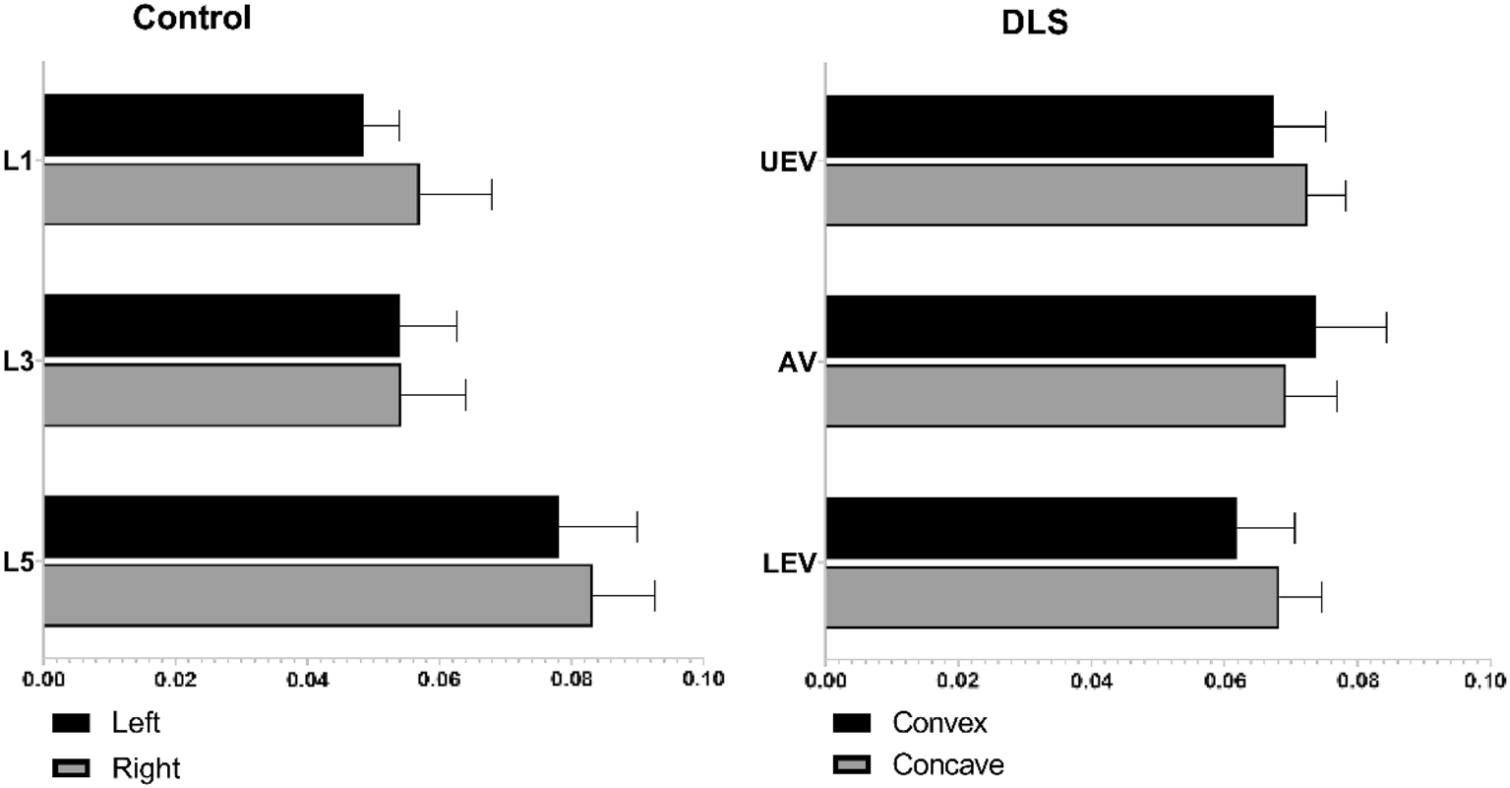
sEMG activity of PVMs at different levels in the control and DLS groups during the static standing phase.

**Figure 7:**
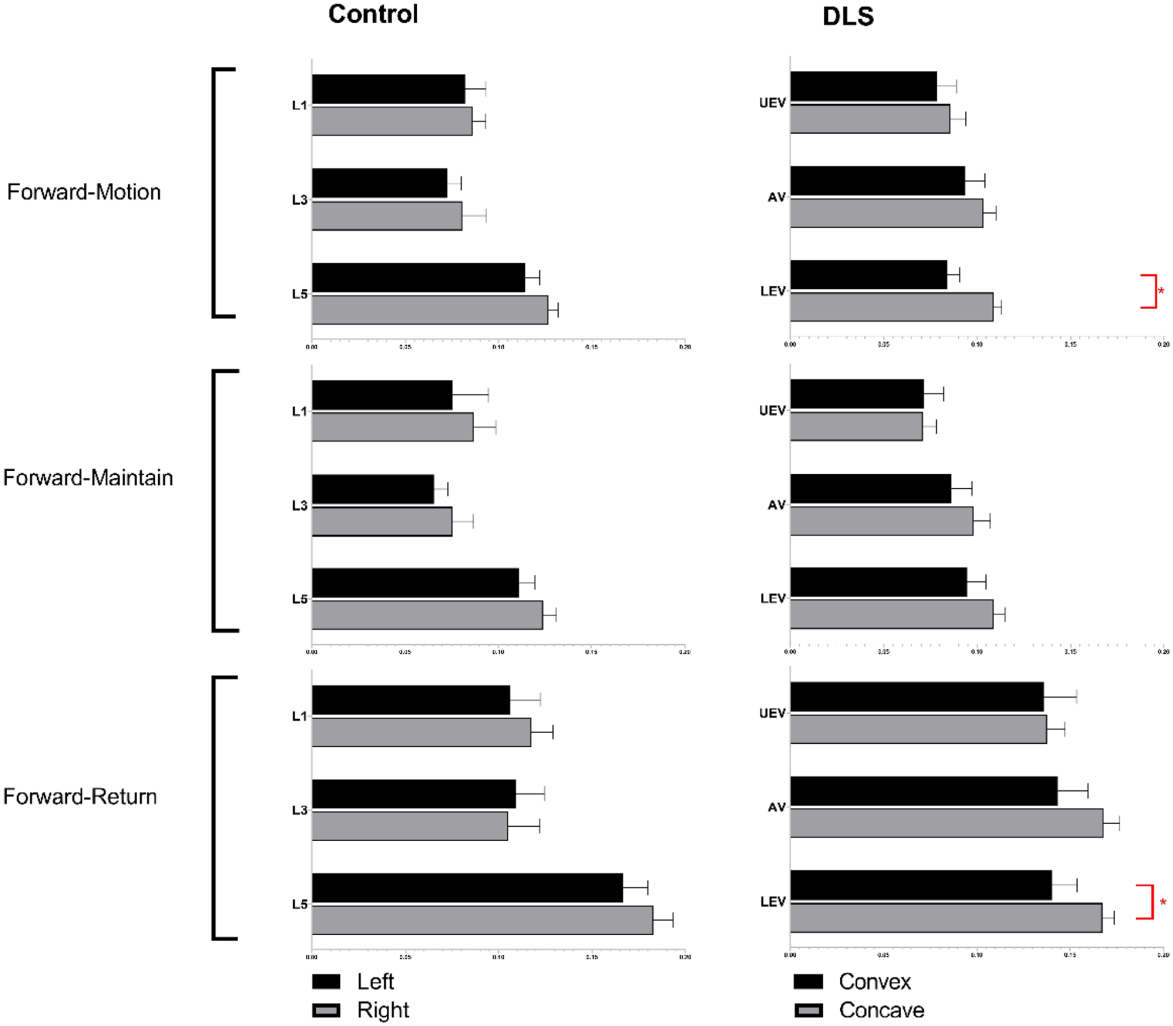
sEMG activity of PVMs at different levels in the control and DLS groups in the standing forward flexion task, *Significant difference in the sEMG activity of PVMs between the convex and concave sides.

**Figure 8:**
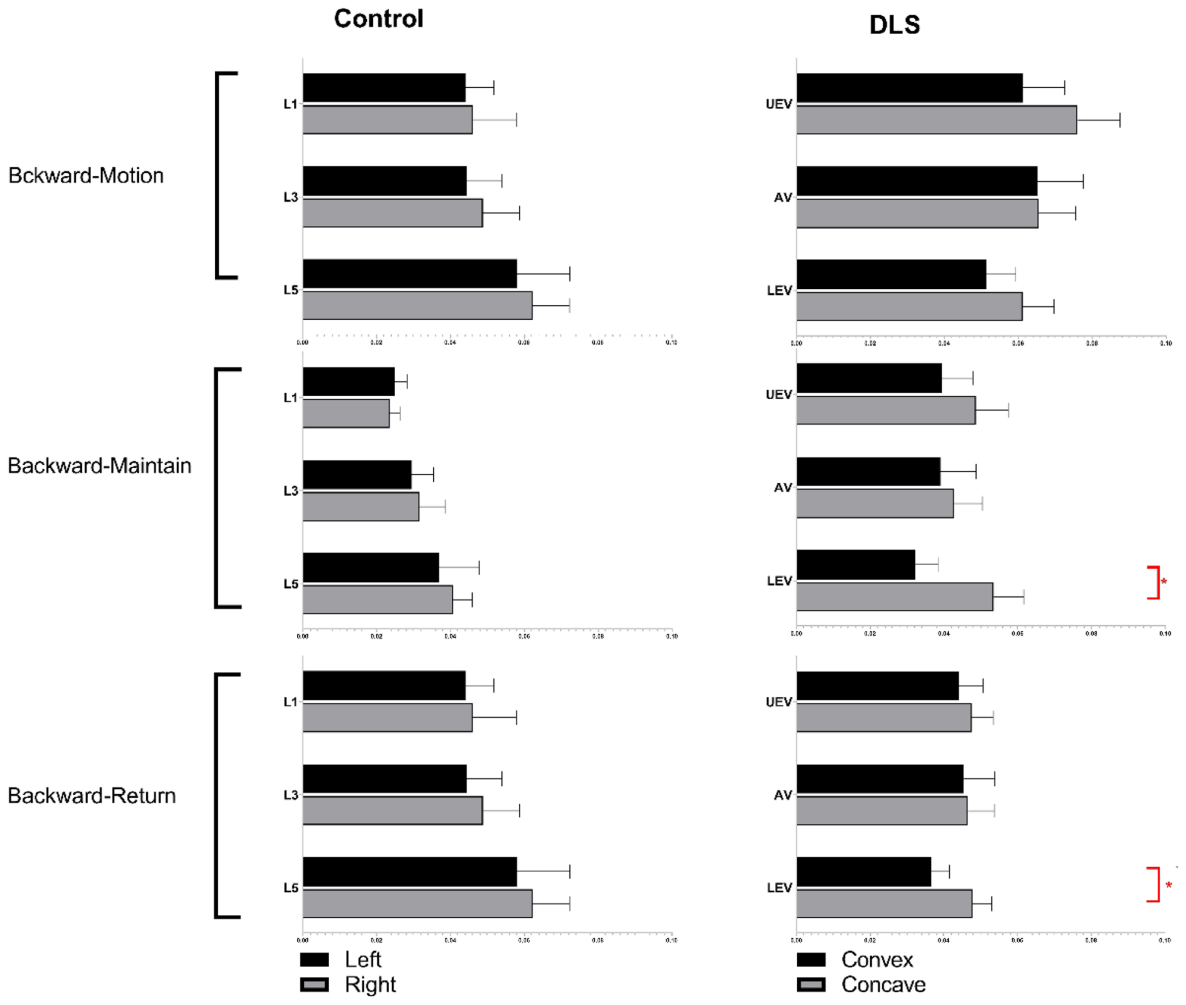
sEMG activity of PVMs at different levels in the control and DLS groups in the standing backward extension task. *Significant difference in sEMG activity of PVMs between the convex and concave sides.

**Figure 9:**
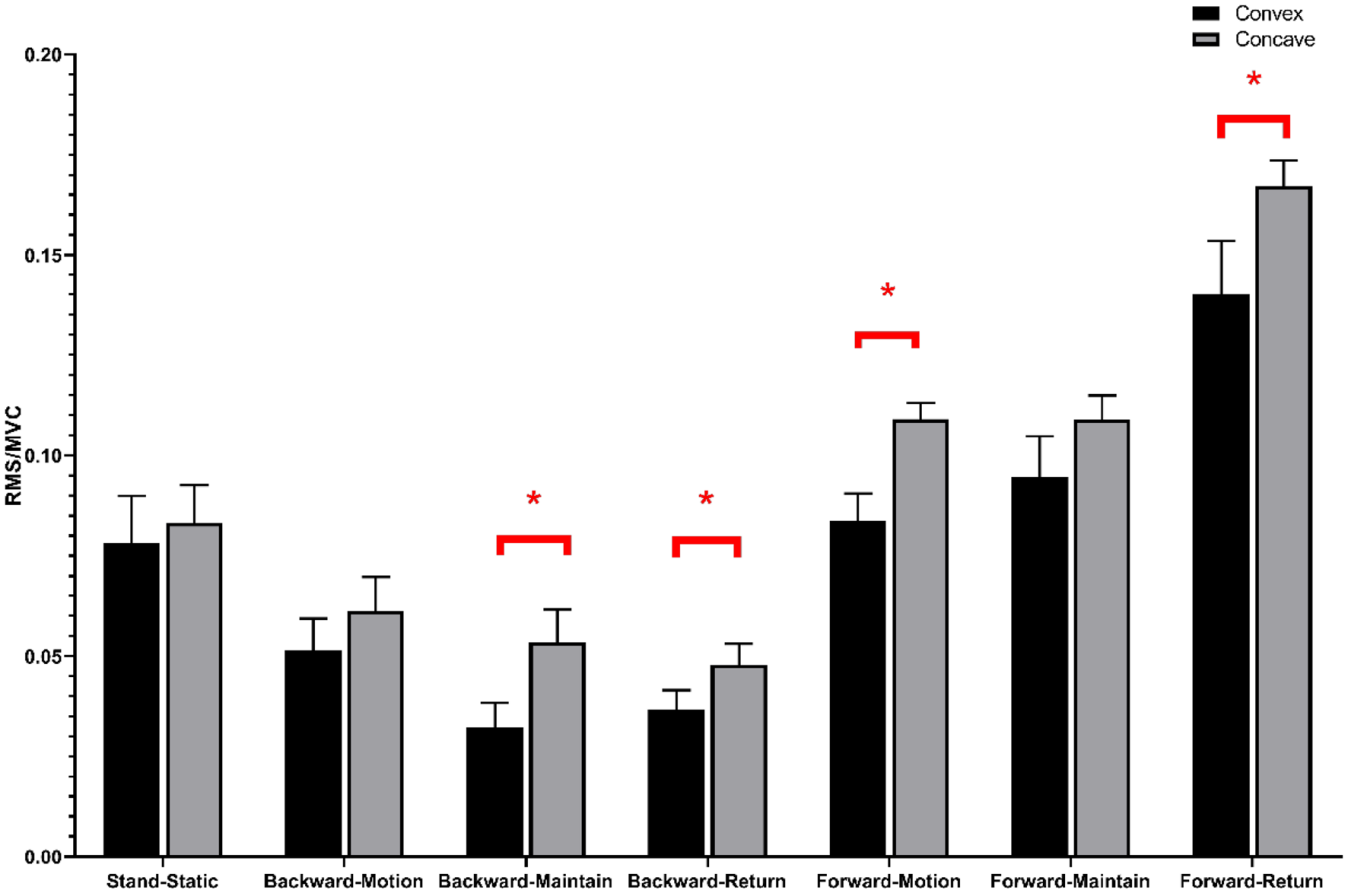
sEMG activity of PVMs at the LEV level in the DLS group in all movement tasks. *Significant difference in sEMG activity of PVMs between the convex and concave sides.

**Figure 10:**
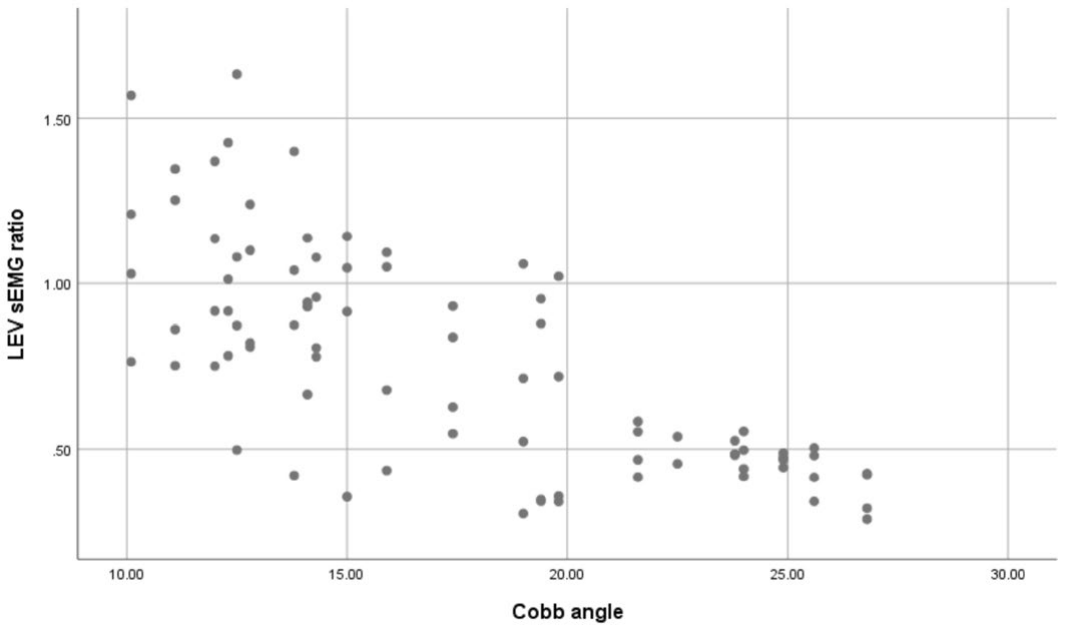
Correlation of the convex/concave sEMG ratio (sEMG activity on the convex side divided by sEMG activity on the concave side) at the LEV level and the Cobb angle in the DLS group during the differential movement phase.

**Figure 11:**
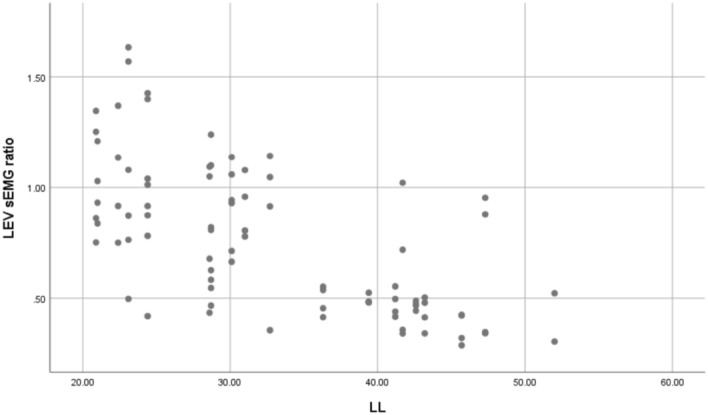
Correlation of the convex/concave sEMG ratio at the LEV level and the LL angle in the DLS group during the differential movement phase.

1. In the static standing phase, there was no difference in the sEMG activity of the PVMs between the left and right sides in the control group, and there was no difference in the sEMG activity of the PVMs between the convex and concave sides in the DLS group (Fig. 6).
2. In the standing forward flexion task, there was no difference in sEMG activity between the left and right sides in the control group, no difference in sEMG activity between the convex and concave sides in the DLS group at the UEV or AV level, and a significant difference in sEMG activity between the convex and concave sides in the DLS group at the LEV level during the motion phase of the standing forward flexion task (t = − 4.38, P = 0.001) and the return phase of the standing forward flexion task (t = − 2.66, P = 0.015) (Fig. 7).
3. In the standing backward extension task, there was no difference in sEMG activity between the left and right sides in the control group, no difference in sEMG activity between the convex and concave sides in the DLS group at the UEV or AV level, and a significant difference in sEMG activity between the convex and concave sides in the DLS group at the LEV level during the maintenance phase of the standing backward extension task (t = − 4.00, P = 0.001) and the return phase of the standing backward extension task (t = − 2.828, P = 0.01) (Fig. 8).
4. During all phases of the movement tasks, in the DLS group, the sEMG activity of the PVMs on the concave side at the LEV level was consistently greater than that on the convex side (Fig. 9).
5. During the phases of movement tasks in which there were differences in sEMG activity (the motion phase of the standing forward flexion task, the return phase of the standing forward flexion task, the maintenance phase of the standing backward extension task, and the return phase of the standing backward extension task), there was a negative correlation between the convex/concave sEMG ratio at the LEV level and the Cobb angle (Fig. 10) and LL angle (Fig. 11).

**Table 2:** sEMG activity (RMS/MVC) of PVMs in the control and DLS groups in the three movement tasks. Significant values are in bold.

Movement task	Electrode level	Control-left	Control-right	P value	Electrode level	DLS-convex	DLS-concave	P value
Stand static	L1	0.049 ± 0.018	0.057 ± 0.036	0.526	UEV	0.067 ± 0.034	0.072 ± 0.026	0.534
	L3	0.054 ± 0.029	0.05 ± 0.032	0.980	AV	0.074 ± 0.046	0.069 ± 0.034	0.579
	L5	0.078 ± 0.039	0.083 ± 0.031	0.451	LEV	0.06 ± 0.0380	0.068 ± 0.028	0.353
Forward-motion	L1	0.082 ± 0.039	0.086 ± 0.024	0.717	UEV	0.078 ± 0.049	0.086 ± 0.039	0.397
	L3	0.073 ± 0.026	0.081 ± 0.044	0.440	AV	0.094 ± 0.051	0.104 ± 0.030	0.404
	L5	0.115 ± 0.026	0.127 ± 0.018	0.246	LEV	0.084 ± 0.032	0.109 ± 0.019	**0.000**
Forward-maintain	L1	0.076 ± 0.067	0.087 ± 0.041	0.558	UEV	0.071 ± 0.051	0.071 ± 0.035	0.953
	L3	0.065 ± 0.026	0.075 ± 0.040	0.366	AV	0.086 ± 0.053	0.098 ± 0.042	0.263
	L5	0.111 ± 0.029	0.124 ± 0.026	0.294	LEV	0.095 ± 0.048	0.109 ± 0.028	0.143
Forward-return	L1	0.106 ± 0.056	0.117 ± 0.040	0.567	UEV	0.136 ± 0.082	0.138 ± 0.0468	0.884
	L3	0.110 ± 0.052	0.105 ± 0.056	0.597	AV	0.143 ± 0.075	0.168 ± 0.040	0.154
	L5	0.167 ± 0.046	0.183 ± 0.036	0.131	LEV	0.140 ± 0.063	0.167 ± 0.030	**0.015**
Backward-motion	L1	0.044 ± 0.026	0.046 ± 0.039	0.866	UEV	0.061 ± 0.051	0.076 ± 0.053	0.256
	L3	0.044 ± 0.032	0.049 ± 0.033	0.633	AV	0.065 ± 0.056	0.066 ± 0.045	0.968
	L5	0.058 ± 0.047	0.062 ± 0.033	0.559	LEV	0.051 ± 0.037	0.061 ± 0.039	0.114
Backward-maintain	L1	0.024 ± 0.011	0.024 ± 0.009	0.743	UEV	0.039 ± 0.038	0.049 ± 0.040	0.216
	L3	0.030 ± 0.019	0.032 ± 0.023	0.793	AV	0.039 ± 0.044	0.043 ± 0.036	0.286
	L5	0.037 ± 0.036	0.041 ± 0.017	0.616	LEV	0.032 ± 0.028	0.054 ± 0.037	**0.001**
Backward-return	L1	0.042 ± 0.019	0.041 ± 0.034	0.945	UEV	0.044 ± 0.030	0.048 ± 0.027	0.552
	L3	0.048 ± 0.033	0.052 ± 0.038	0.727	AV	0.046 ± 0.038	0.047 ± 0.033	0.750
	L5	0.050 ± 0.037	0.052 ± 0.021	0.800	LEV	0.037 ± 0.023	0.048 ± 0.023	**0.010**

The Cobb angle has a statistically significant effect on the convex/concave sEMG ratio (sEMG activity on the convex side divided by sEMG activity on the concave side) at the LEV level, F = 93.791, P < 0.001; the Cobb angle can explain 52.8% of the sEMG ratio, with a moderate degree of influence (Adjust R^2^ = 52.2%). The regression equation is: the sEMG ratio at the LEV level = 1.572 + (− 0.046 × Cobb angle), which is a negative correlation.

The LL angle has a statistically significant effect on the convex/concave sEMG ratio (sEMG activity on the convex side divided by sEMG activity on the concave side) at the LEV level, F = 65.564, P < 0.001; the LL angle can explain 43.8% of the sEMG ratio, with a moderate degree of influence (Adjust R^2^ = 43.2%). The regression equation is: the sEMG ratio at the LEV level = 1.563 + (− 0.024 × LL angle), which is a negative correlation.

## Discussion

Our research found that the sEMG characteristics of PVMs in DLS patients were significantly different from those in healthy controls, which did not show the asymmetry between the left and right sides. In comparison with previous studies, the sEMG characteristics of the PVMs in DLS patients were significantly different from those in AIS patients, but were consistent with the degenerative features in imaging and histology.

1. Asymmetrical sEMG activity in DLS patients appeared at the LEV level, with no difference at the UEV or AV level.

Imaging and histological studies have shown that the rate of muscle degeneration varies in different regions of the lumbar spine, and the degree of PVM degeneration varies between the convex and concave sides at the same level. The degeneration of PVMs was more obvious in the dorsal extensor group, which began at the level of the lower lumbar vertebra and gradually developed upward^17^. Hyun^18^ found that the degree of degeneration and fat infiltration (FI) of PVMs at the lower intervertebral disc level was more serious than that at the upper intervertebral disc level. Xia^19^ found that the relative cross-sectional area (CSA) of PVMs decreased gradually from top to bottom, which was consistent with the results reported by Park^20^. Compared with the upper lumbar level, the lower lumbar level had a higher degree of PVM degeneration.

The appearance of this degeneration pattern has been suggested to be a result of biomechanical compensation. The closer the PVM is to the lower intervertebral disc, the greater the forces it is subjected to, and the more likely the muscle is to degenerate, as evidenced by a gradual increase in muscle FI and muscle degeneration along with the level of the paravertebral extensors from top to bottom^21^.

Our findings are consistent with this degenerative pattern. But in AIS patients, sEMG asymmetry between the convex and concave sides is obvious at the AV level^22–24^. Compared with AIS patients, DLS patients exhibit asymmetrical sEMG activity at the LEV level, demonstrating that the asymmetry is caused by muscle degeneration, whereas the abnormal activity of PVMs on the convex side in AIS patients is considered to be a secondary compensatory response to the scoliosis deformity and therefore appears at the AV level.

2. In DLS patients, the sEMG activity of PVMs on the concave side is more active than that on the convex side at the LEV level.

At present, domestic and foreign scholars believe that the degeneration of PVMs is characterized by a decrease in muscle volume and an increase in fat infiltration^25^. In DLS patients, the degeneration of PVMs at the same level is asymmetrical, and the degeneration on the concave side is greater than that on the convex side, which manifests on imaging and histology. The CSA of PVMs on the convex side is larger than that on the concave side, indicating hyperplasia and hypertrophy; the CSA of PVMs on the concave side is decreased and accompanied by an increased FI^4^, whereas in normal subjects, there is no such asymmetry.

Xie^26^ found that the FI on the concave side multifidus muscle was significantly higher than that on the convex side multifidus muscle in patients with degenerative scoliosis, while the muscle CSA was smaller than that on the convex side. This is similar to the research results reported by Ding^27^. Shafaq^3^ studied histological changes in PVMs in DLS patients and found that the diameter of muscle fibers and the number of nuclei in the multifidus muscle were decreased on the concave side, suggesting that the degeneration of PVMs is more common and severe on the convex side.

The appearance of this asymmetry in degeneration is thought to be the result of biomechanical compensation. Due to the large load carried by the lumbar dorsal extensor group, scoliosis leads to different forces on each side, and the tension load on the convex side is greater than that on the concave side^26,28^. The high load on the convex side causes compensatory hyperplasia and hypertrophy and inhibits adipocyte differentiation^25^, while the concave side shows atrophy and steatosis. Thus, compensatory hypertrophy of the PVMs on the convex side occurs to compensate and maintain the imbalance in the coronal position of the spine and reduce the inclination of the spine to the concave side, while muscle atrophy on the concave side may be associated with increased FI.

We chose RMS/MVC as an indicator of sEMG activation, which produced a higher value in atrophied or hypertrophic muscles, followed by fatigued muscles and a minimum in normal muscles. In PVMs at the same level, weak or heavily degenerated muscles produce greater sEMG activity^29,30^. The results of this study show that the sEMG activity is greater on the concave side than on the convex side in DLS patients, which is consistent with imaging and histological studies. That is, the degree of degeneration is greater on the concave side than the convex side, which also supports the view that biomechanical compensation leads to asymmetrical degeneration on the convex and concave sides.

3. Relationship between sEMG asymmetry on the convex and concave sides and bone structural parameters.

In the sEMG study of AIS patients, the convex/concave sEMG ratio was positively correlated with the Cobb angle; that is, the larger the Cobb angle was, the greater the difference in sEMG activity between the convex and concave sides^10,23^. However, for DLS patients, there have been no reports on the correlation of sEMG asymmetry with bone structural parameters.

Yagi^5^ found that asymmetry in the CSA between the convex and concave sides in DLS patients was correlated with the Cobb angle in lumbar scoliosis. This is similar to the findings reported by Tang^25^, Ding^27^, and Xia^19^. Xie^26^ found that the asymmetry of PVM imaging indexes between the convex and concave sides was not only related to the Cobb angle but also had a weak negative correlation with the LL angle. Therefore, the asymmetry of PVMs in DLS patients reflects the severity of coronal and sagittal imbalance in the lumbar spine^27^.

The larger the Cobb angle is, the greater the asymmetric mechanical load on the concave and convex sides and the worse the stability of the spine, which is the main reason for the aggravation in PVM asymmetry^26^. Our results showed that the convex/concave sEMG ratio in DLS patients was negatively correlated with the Cobb angle and LL angle; that is, the convex/concave sEMG ratio decreased as the Cobb angle and LL angle increased. The reason for this analysis is that the degeneration of PVMs on the convex and concave sides increases with increasing Cobb angle and LL angle in DLS patients, with greater degeneration on the concave side than on the convex side. Muscle activation on the concave side requires higher EMG activity, and the convex/concave sEMG ratio decreases. PVM asymmetry between the convex and concave sides in DLS patients is mainly due to serious degeneration of the concave side. This is different from what occurs in AIS patients, where asymmetry results from excessive muscle loading on the convex side, and more active muscle activity is needed on the convex side to correct the deformity^31^. This correlation explains why dynamic soft tissue stabilization plays a key role in maintaining spinal alignment and PVM misalignment is related to coronal/sagittal imbalance in DLS patients.

## Limitations

1. Considering the presence of EMG crosstalk, changes in the sEMG activity of specific muscles were not measured according to the anatomical structure, and the sEMG characteristics of scoliosis were measured at only three levels (UEV, AV, and LEV).
2. There were differences in sEMG activity among individuals in the groups, which may have been caused by variations in muscle mass, body posture, and electrodeposition.
3. The asymmetry of sEMG activity at the LEV level does not appear in all phases of movement tasks, which may due to the large variation in movement amplitude from one movement task to another and the different degrees of mobilization of the PVMs.

## Conclusion

1. In DLS patients, asymmetrical sEMG activity between the convex and concave sides appears at the LEV level, with more sEMG activity on the concave side than on the convex side, which is significantly different from that observed in healthy controls and AIS patients. This asymmetrical sEMG activity in PVMs is consistent with the findings of imaging and histological studies.
2. In DLS patients, the main reason for the asymmetrical sEMG activity between the convex and concave sides is muscle degeneration, which is predominant on the concave side; this is significantly different from the findings in AIS patients.
3. Asymmetrical sEMG activity between the convex and concave sides in DLS patients is related to the Cobb angle and LL angle.

## Data Availability

The datasets used and/or analyzed during the current study are available from the corresponding author on reasonable request.

## Abbreviations

DLS: Degenerative lumbar scoliosis
AIS: Adolescent idiopathic scoliosis
PVM: Paravertebral muscle
sEMG: Surface electromyography
IMU: Inertial measurement unit
LL: Lumbar lordotic angle
UEV: Upper end vertebra
AV: Apical vertebra
LEV: Lower end vertebra
FI: Fat infiltration
CSA: Cross-sectional area
